# Clinical Therapeutic Effects of the Application of Doxycycline in the Treatment of Periodontal Disease

**DOI:** 10.3889/oamjms.2016.021

**Published:** 2016-01-22

**Authors:** Spiro Spasovski, Zlatanka Belazelkoska, Mirjana Popovska, Aneta Atanasovska-Stojanovska, Vera Radojkova-Nikolovska, Ilijana Muratovska, Natasa Toseska-Spasova, Biljana Dzipunova, Bruno Nikolovski

**Affiliations:** 1 *PHO „Fjolla Medika”, Skopje, Republic of Macedonia*; 2 *Faculty of Dentistry, Ss. Cyril and Methodius University of Skopje, 1000 Skopje, Republic of Macedonia*; 2 *PHO ETERNAdent, Skopje, Republic of Macedonia*

**Keywords:** periodontal disease, doxycycline, sub-dose, gingival inflammation, gingival bleeding

## Abstract

**OBJECTIVE:**

To compare the therapeutic effects of the application of doxycycline-full dose (100 mg) and sub-dose (20 mg) in the treatment of periodontal disease.

**MATERIAL AND METHODS:**

A total of 60 patients with periodontal disease were examined. Patients are divided into two groups: A) treated with antimicrobial dose of 100 mg doxycycline once daily for 30 days, and B) treated with 2 x 20 mg/day. doxycycline, during 75 days. Among all patients a conservative treatment was carried out and ordinated the proper dose doxycycline in total dose during treatment from 3 gr. Index of dental plaque by Löe-Sillness, index of gingival inflammation and gingival bleeding by Cowell were followed.

**RESULTS:**

Values of dental plaque in relation first examination, 10th, 20th day, 1 month and 2.5 months, showed that after 2.5 months, average value (x = 0.83) of dental plaque in second group is slightly less than the value (x = 0.93) of dental plaque in the first group. The average value (x = 0.17) of gingival inflammation in second group is significantly less than the value (x = 0.50) of gingival inflammation in the first group. The average value (x = 0.97) of gingival bleeding in patients from the first group was significantly higher than value(x = 0.37) of gingival bleeding in the second group.

**CONCLUSION:**

Patients whose therapy was helped by a sub-dose doxycycline demonstrated positive therapeutic effects on gingival inflammation and bleeding.

## Introduction

Treatment of periodontal disease includes removing of dental plaque and other irritating factors (conservative treatment) or performing surgical methods, which sometimes include the use of antibiotic therapy administered systemically or locally [1, 2]. The administration of local and systemic antibiotics, anti-inflammatory drugs, or sub-antimicrobial small doses of doxycycline applied as adjuvants have the opportunity to provide additional positive therapeutic effects [3, 4]. Sub-antimicrobial dose of doxycycline is 20 mg dose of the antibiotic applied twice daily in the treatment of periodontal disease. It is believed that a dose of 20 mg provides quite effective inhibition of enzymes, cytokines and osteoclasts than any antibiotic that would be applied in full or normal dose. On the other hand research studies have not spotted noticeable changes in the oral flora or bacterial flora in other organs in the individual’s body because of which they identified as extremely recommended dosage which is a serious clinical utility when applied only in addition to periodontal pockets debridement [3]. For the clinician, this is particularly favorable moment, which enables broad applicability.

Well known fact is that periodontal disease is a disease known as infection of the periodontal tissue complex. The primary initiation belongs to the bacteria which alter local immune response and result in appropriate tissue destruction. Due to the infectious nature of the disease the main aim of treatment should be suppression of the effective bacterial populations responsible for the dissolution of the periodontal tissues [5, 6]. Recent findings show that the local causal therapy conducted in the affected periodontium is not always sufficient to eliminate bacteria, so under certain conditions in the overall therapeutic treatment, systemic treatment with antibiotics may be included [6-8].

Bacterial plaque is a major factor for the initiation of chronic periodontitis (CP), but characteristic clinical signs (periodontal pocket depth, epithelial apical migration and alveolar bone destruction) primarily are a result of disrupted and insufficient immune and defense mechanisms of the host [9]. The success of the results varies between individuals and depends on the production and the level of proinflammatory mediators, enzymes and cytokines in all development stages of the disease [10].

The key between those two extremes are matrix metalloproteinasis (MMPs), a family of proteolytic enzymes released by macrophages, tissue fibroblasts, epithelial cells and neutrophils (PMNs). Damage of the collagen, the major structural protein in periodontium has his part in the destruction of the epithelial attachment and bone resorption [10]. LDH which helps the destructive and resorptive processes in periodontal tissue complex joins on these pathogenetic mechanisms of chronic periodontitis.

Considering these facts we have set the goal of this paper: to compare the therapeutic effects of the application of doxycycline - from the full dose (100 mg) and sub-dose (20 mg) in the treatment of periodontal disease.

## Material and Methods

The choice of patients that are part of the study is made by chance, and is based on a personal position on each of them. Of those who voluntarily showed a wish to be part of the study, is provided written consent for joining in the investigation. In the selection of patients are taken into consideration the criteria for inclusion and exclusion of the study, according to the protocol approved by the Ethic Commission of Faculty of Dentistry at UKIM in Skopje.

The study was performed of 60 patients in age from 30-70 years with clinically manifested mild form of chronic periodontitis where depth of periodontal pockets on certain representative group of teeth was estimated at 3-5 mm. The diagnosis of each individual of the study group was set according collected anamnestic data which also includes dental and medical history of the patients, clinical examinations and analysis the X-ray image.

The selection of patients is made at the Clinic of oral pathology and periodonthology, at the University of Dental Clinical Centre in Skopje. As additional criterias are taken into account were the criteria for inclusion and exclusion of the study:

### Criteria for inclusion

patients with periodontal disease which manifest the periodontal pockets depth of 3-5 mm; patients who in the last two months are not receiving drugs from any group of antibiotics; patients with at least three teeth in one side of the jaw; and patients who have at least 12 teeth in the mouth.

### Criteria for exclusion

patients receiving systemic antimicrobial drugs; patients receiving non-steroidal anti-inflammatory drugs; patients with diagnosed any cardiac, renal or acute infectious illness; patients in whom not conducted curettage in periodontal pockets in the last 3 months; smokers, alcoholics, pregnant women, patients with brest cancers; patients allergic to antibiotics; patients with poor oral hygiene; patients who are using any herbal drugs and extracts.

Patients are divided into two groups (total n = 60). Group A is treated with antimicrobial dose of 100 mg doxycicline capsule (30 patients) once daily in progress in 30 days, and in group B with the same number of subjects where the ordinated dose were 2 x 20 mg doxycicline capsule daily for 75 days.

In all subjects, after the diagnostics and determining the condition, there were conducted conservative treatments which mean removal of dental calculus, soft deposites, and dental plaque and other iritative factors, scaling and root planing. After that, there was administrated doxycycline in the appropriate dose (depending on the group to which it is intended).

The total dose that the patients received in the course of therapeutic treatment without distinction of dosage and dynamics of applications was 3 grams. The first group has the systemic administration of doxycycline 100 mg. once daily, and total planned dose received within 30 days. The second group of patients who received doxycycline twice by 20 mg daily (in the morning and evening) ended this therapy after 75 days, also in total dose of 3 g.

### Patients were conducted on following clinical research

Index of dental plaque, according to Löe-Sillness; Index on gingival inflammation and gingival bleeding according Cowell.

The index of dental plaque, according to Löe-Sillness was registered with visual method through coloring with methylene blue, according to which was the numbered index which belongs to.

The index of gingival inflammation and gingival bleeding index according Cowell were measured separately.

The inflammation was determined with inspection, and for gingival bleeding was applied the method of sonding, too.

The statistical data processing was performed in a statistical program STATISTICA 7.1. The differences in the values of the dental plaque, gingival inflammation, gingival bleeding, periodontal pockets depth, clinical attachment level, overview on 10^th^ day, 20^th^ day, 1 month and after 75 days, were tested with Friedman ANOVA test (ANOVA Chi Sqr.)

The differences in the values of the dental plaque, gingival inflammation and gingival bleeding between the two subgroups of patients (patients who received doxycicline 2 x 20 mg daily 2.5 months and patients who received doxycicline 100 mg daily for 30 days) were tested by Mann-Whitney U test (Z).

## Results

Differences in values of dental plaque in the relation: first examination, 10^th^ day, 20^th^ day, 1 month and 2.5 months, as a result of the applied therapy in both groups are shown on Table 1. At the first examination for Z = 0.00 and p < 0.05 (p = 0.99) there was no significant difference in the average value of the dental plaque in both groups.

**Table 1:** The difference between the investigated groups according to the received value of dental plaque in all investigated time intervals

Dental plaque	Rank Sum 1 x 100 mg	Rank Sum 2 x 20 mg	U	Z	p-level	Valid N 1 x 100 mg	Valid N 2 x 20 mg
First examin	914.00	915.00	450.00	0.00	0.99	30	30
10th day	702.00	1128.00	237.00	-3.15	**0.002**	30	30
20 th day	930.00	900.00	435.00	0.22	0.82	30	30
1 month	885.00	945.00	420.00	-0.44	0.66	30	30
2.5 months	957.50	872.50	407.50	0.63	0.53	30	30

After 10 days from the applied therapy the average value of dental plaque (x = 0.97) in patients from the second group (doxycycline 2 x 20 mg/2.5 months) for Z = -3.15 and p < 0.01 (p = 0.002) was significantly higher than the average value of the in dental plaque (x = 0.37) in patients from the first group (doxycycline100 mg/30 days).

After 20 days from the applied therapy the average value (x = 0.43) on dental plaque in patients from the second group (doxycycline 2 x 20 mg/2.5 months) for Z = 0.22 and p> 0.05 (p = 0.82) is only slightly lower than the average value (x = 0.47) of dental plaque in patients from the first group (100 mg doxycycline/30 days).

After 1 month of the applied therapy the average value (x = 0.33) of dental plaque in patients from the second group (doxycycline 2 x 20 mg/2.5 months) for Z = -0.44 and p > 0.05 (p = 0.66) was only slightly higher than the average value of the (x = 0.27) in dental plaque in patients from the first group (doxycycline100 mg/30 days).

After 2.5 months, the average value (x = 0.83) of dental plaque in patients of the second group (doxycycline 2 x 20 mg/2.5 months) for Z = 0.63 and p < 0.05 (p = 0.53) unsignificantly is less than the average value (x = 0.93) of dental plaque in patients from the group A (doxycycline 100 mg/30 days) (Table 1).

Mean differences between index of gingival inflammation in relation with first check up, 10^th^ day, 20^th^ day, one month and 2.5 month later, as a result of applied therapy in both examined groups, are shown in Table 2.

**Table 2:** Difference between examined groups according to the obtained values of index of gingival inflamation between all tested intervals

Gingival inflamation	Rank Sum 100 mg	Rank Sum 2 x 20 mg	U	Z	p-level	Valid N 100 mg	Valid N 2 x 20 mg
First examin	942.50	887.50	422.50	0.41	0.68	30	30
10th day	1092.00	738.00	273.00	2.62	**0.009**	30	30
20th day	915.00	914.00	450.00	0.00	0.99	30	30
1 month	914.00	915.00	450.00	0.00	0.99	30	30
2.5 months	1065.00	765.00	300.00	2.22	**0.06**	30	30

On the first check up mean value (x = 1.57) of gingival inflammation was slightly greater than the average value in patient from second group x = 1.47), Z = 0.41 and p > 0.05 (p = 0.68).

Ten days after applied therapy average values of gingival inflammation in patient from the first group (x = 0.83) was significantly greater than the average values in second group (x = 0.40), Z = 2.62 and p < 0.01 (p = 0.009).

Twenty days after applied therapy there are not significantly differences in the average values of index of gingival inflammation between examined groups, Z = 0.00 and p > 0.05 (p = 0.99).

One month after applied therapy there are not significantly differences in the average values of index of gingival inflammation between examined groups, Z = 0.00 and p > 0.05 (p = 0.99).

On the check up two and a half months after applied therapy average values conducted in patients from second group were significantly smaller than the consequently values in patient from the first group, Z = 2.22 and p > 0.05 (p = 0.03) (Table 2).

Mean differences between index of bleeding of probing BOP in relation with first check up, 10^th^ day, 20^th^ day, one month and 2.5 month later, as a result of applied therapy in both examined groups, are shown in Table 3.

**Table 3:** Difference between examined groups according to the obtained values of BOP in all tested intervals

Gingival bleeding	Rank Sum 2 x 20 mg	Rank Sum 1 x 100 mg	U	Z	p-level	Valid N 100 mg	Valid N 2 x 20 mg
First examin	914.00	915.00	450.00	0.00	0.99	30	30
10th day	991.00	839.00	374.00	1.12	0.26	30	30
20th day	915.00	914.00	450.00	0.00	0.99	30	30
1 month	914.00	915.00	450.00	0.00	0.99	30	30
2.5 months	679.00	1151.00	214.00	-3.49	**0.000**	30	30

On the first check up there are not any significant differences between two examined groups, Z = 0.00 and p > 0.05 (p = 0.99).

Then days after applied therapy average values on BOP index was slightly greater in patients which consisted second group, but without significantly differences between both examined groups, Z = 1.12 and p > 0.05 (p = 0.26).

Twenty days and one month after applied therapy there are not any significant differences between two examined groups, Z = 0.00 and p > 0.05 (p = 0.99).

On the check up two and a half months after applied therapy average values conducted in patients from second group were significantly smaller than the consequently values in patient from the first group, Z = -3.49 and p < 0.001 (p = 0.000) (Table 3).

## Discussion

Today there are a lot of antibiotics which may be ordained, locally or systemically [11]. Tetracyclines are one of the antibiotics that have a broad spectrum of activity and are able to inhibit the anaerobic microorganisms. The literature shows that it is possible to happen in a very short period, usually 5 days [12]. It has been proven that doxycycline is the most common antibiotic that belongs to the group of antimicrobials that are active against most periopatogens due to low minimum inhibitory concentration (MIC) [13].

In our study are compared clinical effects in patients with chronic periodontitis treated with doxycycline 100 mg. once a day for one month and 20 mg doxycycline twice daily in period of 2.5 months.

It is evident from the study, that despite the first clinical examination in patients from both groups, all index values recorded improved clinical findings whether it is a dose of 100 mg or 40 mg daily.

Descriptive statistics of index of dental plaque according to Löe-Sillness at patients undergoing systemic application of doxycycline 100 mg once daily for 30 days, showed variation and systematic reduction from the first examination to period of 75 days. The differences between the values of dental plaque in relation to first examination, 10th, 20th day, 1 month and 2.5 months for ANOVA Chi Sqr. = 81.00 and p < 0.001 (p = 0.000) there is significant difference between the values of dental plaque in analyzed relation. We get almost identical findings with gingival inflammation and gingival bleeding.

The average value of gingival bleeding (x = 0.93) after 2.5 months Z = 3.30 and p <0.001 (n = 0.000) is significantly lower than the average value of gingival bleeding (x = 1.53) at the first examination.

From the obtained findings it is quite certain that improved clinical effects were obtained after applying doxycycline in dose 2 x 20 mg. Differences in values of dental plaque in relation first examination, 10th, 20th day, 1 month and 2.5 months, as a result of the applied therapy in both groups shows that after 2.5 months average value of dental plaque (x = 0.83) in patients in the second group (2 x 20 mg doxycycline/2.5 months) for Z = 0.63 and p > 0.05 (p = 0.53) is slightly lower than the average value (x = 0.93) of dental plaque of patients in the first group (doxycycline 100 mg/30 days). Evaluation of all remaining clinical parameters showed definite advantages in the group treated with sub-dose 2 x 20 mg doxycycline. After 2.5 months average value (x = 0.17) of gingival inflammation in patients in the second group (2 x 20 mg.doxycycline/2.5 months) for Z = 2.22 and p > 0.05 (p = 0.03) was significantly lower than the average value (x = 0.50) of gingival inflammation in patients from the first group (doxycycline 100 mg/30 days).

In terms of index of gingival bleeding, after 2.5 months, average value of gingival bleeding (x = 0.97) at patients in the first group (doxycycline 1 x 100 mg daily/2.5 months) and Z = -3.49, p < 0.001 (n = 0.000) was significantly greater than the average value of gingival bleeding (x = 0.37) at patients in the second group (doxycycline 2 x 20 mg/30 days). The rationale for choosing the application of doxycycline in addition to conventionally applied method is supported by its ability to achieve higher concentrations in gingival cervical fluid, and the possibility of modulating the clinical effect on the host. Doxycycline inhibits matrix metalloproteinase’s, which mediates in reduced opportunities for periodontal breakdown, which first manifests itself in reduced gingival inflammation and reduced gingival bleeding [14]. Some studies have failed to find a positive therapeutic effect of doxycycline doxycycline [15-17].

Although in the literature exists quite conflicting results, tetracycline and its related drugs are known to participate in the activation of latent pro-MMP, MMP de-escalation, and neutralizing oxidative mechanisms responsible for periodontal tissue destruction [18, 19]. This evidence provided the basis for a new therapeutic approach to control periodontal disease not only in healthy people, but also in those with compromised medical health, especially among patients with diabetes mellitus for the use of tetracycline and their derivatives in the treatment of periodontal disease [20]. Our findings are consistent with the findings of many authors [4, 21-25].

In this regard, we support the notion that systemic antibiotics administered as adjunctive therapy in sub- antimicrobial dose, may offer an additional advantage over the conducted conventional therapy. In this context primarily refers to the clinical loss of attachment and periodontal pocket depth. However, differences in study methodology and lack of certain facts can be a serious handicap for comprehensive analysis. It was difficult to extract definitive conclusions from the study, although patients with deep pockets, progressive or “active form” of the disease or specific microbial profile, can be beneficial after this additional treatment [26]. In contrast to these findings [27] concluded that the evidence for the additional benefit of adjuvant therapy with antibiotics in patients with chronic periodontitis is insufficient and unconvincing. Based on the obtained findings of the survey we can conclude that in patients whose therapy was supported by a sub-dose doxycycline 2 x 20 mg demonstrated positive clinical therapeutic effects on gingival inflammation and gingival bleeding.
